# Molecular evidence on phenotypic variation in the poorly known acanthocephalan species *Rhadinorhynchus cololabis* Laurs & McCauley, 1964 (Echinorhynchida: Rhadinorhynchidae)

**DOI:** 10.1051/parasite/2025069

**Published:** 2025-11-28

**Authors:** Ke-Yu Wang, Yuan-Yuan Xie, Hui-Xia Chen, Liang Li

**Affiliations:** 1 Hebei Collaborative Innovation Center for Eco‐Environment; Hebei Key Laboratory of Animal Physiology, Biochemistry and Molecular Biology; College of Life Sciences, Hebei Normal University 050024 Shijiazhuang Hebei Province PR China; 2 Ministry of Education Key Laboratory of Molecular and Cellular Biology 050024 Shijiazhuang Hebei Province PR China

**Keywords:** Acanthocephala, Rhadinorhynchidae, DNA taxonomy, Phenotypic variation, Mitochondrial genome, Species partition

## Abstract

Acanthocephalans of the genus *Rhadinorhynchus* parasitize various marine fishes worldwide. However, the true diversity of *Rhadinorhynchus* is still unclear. In this study, we found an example of phenotypic variation in trunk spines of the poorly known rhadinorhynchid species *R. cololabis* Laurs & McCauley, 1964. According to the number and distribution of trunk spines, the present specimens of *R. cololabis* can be divided into two distinct morphotypes, which may erroneously be recognized as distinct taxa in the absence of molecular data. However, the Assemble Species by Automatic Partitioning (ASAP) and Bayesian inference (BI) analyses based on different nuclear and mitochondrial sequence data, all confirm that the two distinct morphotypes are conspecific, and do not represent two separate genetic lineages. Our ASAP and BI results of *cox1* data also suggest that is *R. villalobosi* Martínez-Flores *et al.*, 2025 is a synonym of *R. trachinoti* Grano-Maldonado *et al.*, 2025, and challenge the validity of *R. dorsoventrospinosus* Amin *et al.*, 2011, and *R. hiansi* Soota & Bhattacharya, 1981. The present findings also indicate that the number and distribution of trunk spines vary markedly in some species of *Rhadinorhynchus*, and care must be taken when differentiating *Rhadinorhynchus* species based on this feature. Additionally, the complete mitogenome of *R. cololabis* is presented for the first time, which has only 13,567 bp, and displays a very high level of similarity with the mitogenome of *R. laterospinosus* in both nucleotide sequences (94.6%) and amino acid sequences of 12 protein-coding genes (93.8%). However, comparative mitogenomics support *R. cololabis* and *R. laterospinosus* representing two separate taxa.

## Abstract (fr)

Les Acanthocéphales du genre *Rhadinorhynchus* parasitent divers poissons marins du monde entier. Cependant, la véritable diversité de *Rhadinorhynchus* reste encore peu connue. Dans cette étude, nous avons trouvé un exemple de variation phénotypique des épines du tronc de l’espèce de Rhadinorhynchidae peu connue *R. cololabis* Laurs & McCauley, 1964. D’après le nombre et la répartition des épines du tronc, nos spécimens de *R. cololabis* peuvent être divisés en deux morphotypes distincts, qui pourraient être confondus avec des taxons distincts en l’absence de données moléculaires. Cependant, les analyses ASAP et BI basées sur différentes données de séquences nucléaires et mitochondriales confirment toutes que les deux morphotypes distincts sont conspécifiques et ne représentent pas deux lignées génétiques distinctes. Nos résultats ASAP et BI des données sur *cox1* suggèrent également de traiter *R. villalobosi* Martínez-Flores *et al.*, 2025 comme un synonyme de *R. trachinoti* Grano-Maldonado *et al.*, 2025, et remettent en question la validité de *R. dorsoventrospinosus* Amin *et al.*, 2011, et *R. hiansi* Soota & Bhattacharya, 1981. Les présents résultats indiquent également que le nombre et la distribution des épines du tronc varient considérablement chez certaines espèces de *Rhadinorhynchus*, et que la prudence doit être de mise lors de la différenciation des espèces de *Rhadinorhynchus* sur la base de cette caractéristique. De plus, le mitogénome complet de *R. cololabis* est présenté pour la première fois. Il ne compte que 13 567 pb et présente une très forte similarité avec celui de *R. laterospinosus*, tant au niveau des séquences nucléotidiques (94,6%) que des séquences d’acides aminés de 12 gènes codant pour des protéines (93,8%). Cependant, la mitogénomique comparative suggère que *R. cololabis* et *R. laterospinosus* représentent deux taxons distincts.

## Introduction

The genus *Rhadinorhynchus* Lühe, 1911 is a common group of acanthocephalans containing more than 40 described species that parasitize various marine fishes worldwide [1, 3, 4, 16, 38]. However, the true diversity of *Rhadinorhynchus* remain unclear, because most species in this genus are defined under the morphospecies concept, which does not always yield taxa consistent with the actual biological species in some cases of Acanthocephala [26, 27, 42, 51]. The number and distribution pattern of trunk spines are considered to be key diagnostic characters for traditional species identification of *Rhadinorhynchus* [2, 38]. However, we are still not sure whether it is reliable to use the number and distribution of trunk spines as critical criteria for delineating the species of *Rhadinorhynchus*.

Recently, some studies have proven that nuclear and mitochondrial sequence data, including mitochondrial genomes, play important roles in the detection of cryptic species, assessment of phenotypic variation, identification of cystacanths, and clarification of phylogenetic relationships of acanthocephalans [9, 16, 18, 20, 21, 26–29, 37, 45–47, 49–51]. However, the current genetic database for *Rhadinorhynchus* remains insufficient. To date, a total of 12 species of *Rhadinorhynchus* have been genetically sequenced [4, 5, 7, 8, 12–14, 16, 18, 21, 28–31, 43]. In the family Rhadinorhynchidae, only *R. laterospinosus* Amin, Heckmann & Ha, 2011 has had a complete mitochondrial genome documented [47].

*Rhadinorhynchus cololabis* Laurs & McCauley, 1964 is a poorly known acanthocephalan species, originally described from the Pacific saury *Cololabis saira* (Brevoort) (Beloniformes: Scomberesocidae) off the coast of the United States (Oregon) [23]. Since then, only Motora (2016) [32] recorded this species from *Oncorhynchus masou* (Brevoort) (Salmoniformes: Salmonidae) in the Sea of Japan. During a helminthological survey of Chinese marine fishes, large numbers of *Rhadinorhynchus* specimens identified morphologically as *R. cololabis* were collected from *C. saira* in the South China Sea. Examination of specimens using light and scanning electron microscopy revealed the presence of remarkable phenotypic variation in the number and distribution of trunk spines among different individuals. In order to evaluate whether the present specimens of *R. cololabis*, with a different number and distribution of trunk spines, belong to a complex of sibling species or a single species, Assemble Species by Automatic Partitioning (ASAP) analyses and Bayesian inference (BI) were performed based on different nuclear and mitochondrial sequence data. Furthermore, we also sequenced and annotated the complete mitochondrial genome of this species for the first time, to enrich the mitogenomic data and reveal the pattern of mitogenomic evolution of the family Rhadinorhynchidae.

## Materials and methods

### Ethics approval

This study was conducted under the protocol of Hebei Normal University (LLSC2024090). All applicable national and international guidelines for the protection and use of animals were followed.

### Acanthocephalan collection and morphological observation

A total of 46 individuals of *C. saira* collected from the South China Sea (off Nanning City, Guangxi Zhuang Autonomous Region, China) in 2023, were examined for helminth parasites, and 57 acanthocephalan specimens were isolated from the intestine of 31 fish hosts [prevalence 67.4%, intensity of infection 1–22 (mean = 4.55) specimens]. Specimens were washed using clear water, and then fixed and stored in 80% ethanol for further study.

For light microscopy studies, acanthocephalans were cleared in lactophenol and made in temporary mounts. Photomicrographs were recorded using a Nikon^®^ digital camera coupled to a Nikon^®^ optical microscope (Nikon ECLIPSE Ni-U, Nikon Corporation, Tokyo, Japan). For scanning electron microscopy (SEM), specimens were post-fixed in 1% OsO_4_ (Osmium Tetroxide), dehydrated *via* an ethanol series and acetone, and then critical point dried. The material was coated with gold at about 20 nm and examined using a Hitachi SU8600 scanning electron microscope at an accelerating voltage of 20 kV.

**Figure 1:**
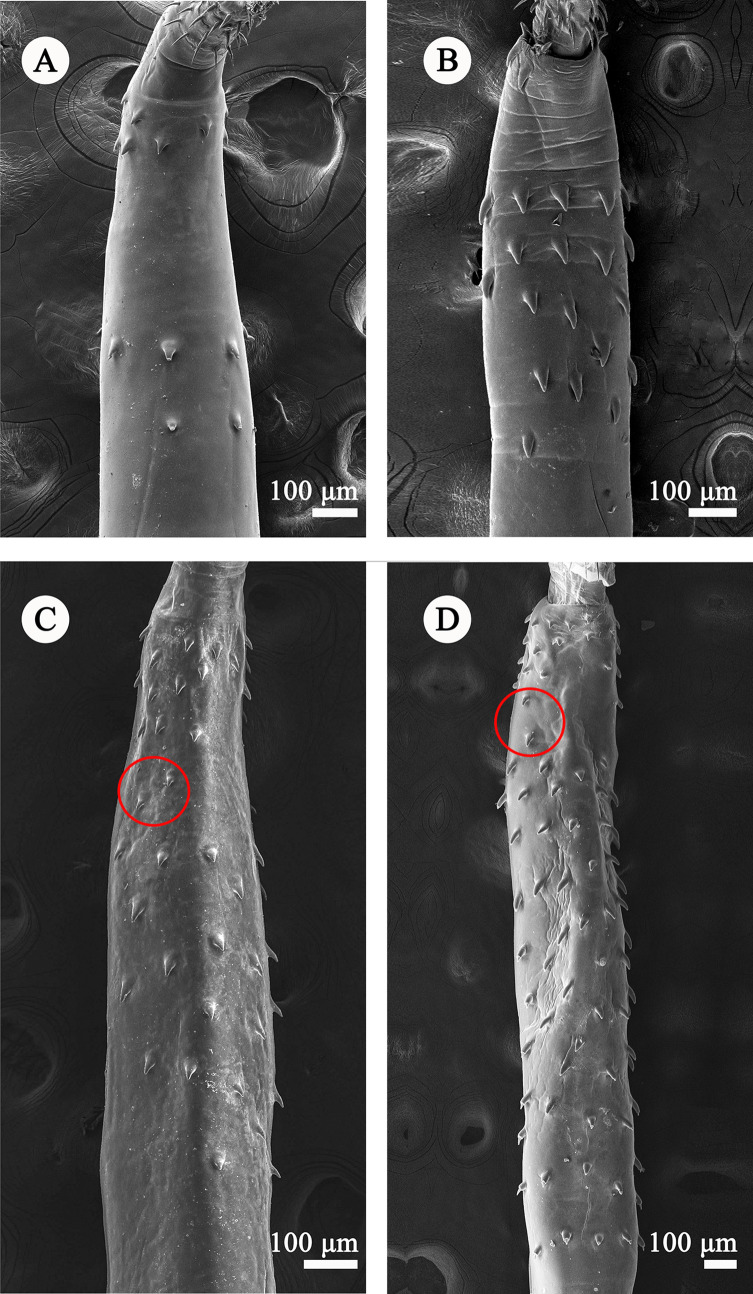
Scanning electron micrographs of two different morphotypes of *Rhadinorhynchus cololabis*. (A) Morphotype I of male: anterior trunk possessing fewer spines, divided by a distinct aspinose zone into anterior and posterior fields, ventral view; (B) Morphotype I of female: anterior trunk possessing fewer spines, divided by a distinct aspinose zone into anterior and posterior fields, ventral view; (C) Morphotype II of male: anterior trunk possessing more spines, lacking distinct aspinose zone (red circle showing two lateral spines connecting the anterior and posterior fields together), ventral view; (D) Morphotype II of female: anterior trunk possessing more spines, lacking a distinct aspinose zone (red circle showing two lateral spines connecting the anterior and posterior fields together), ventral view.

**Figure 2:**
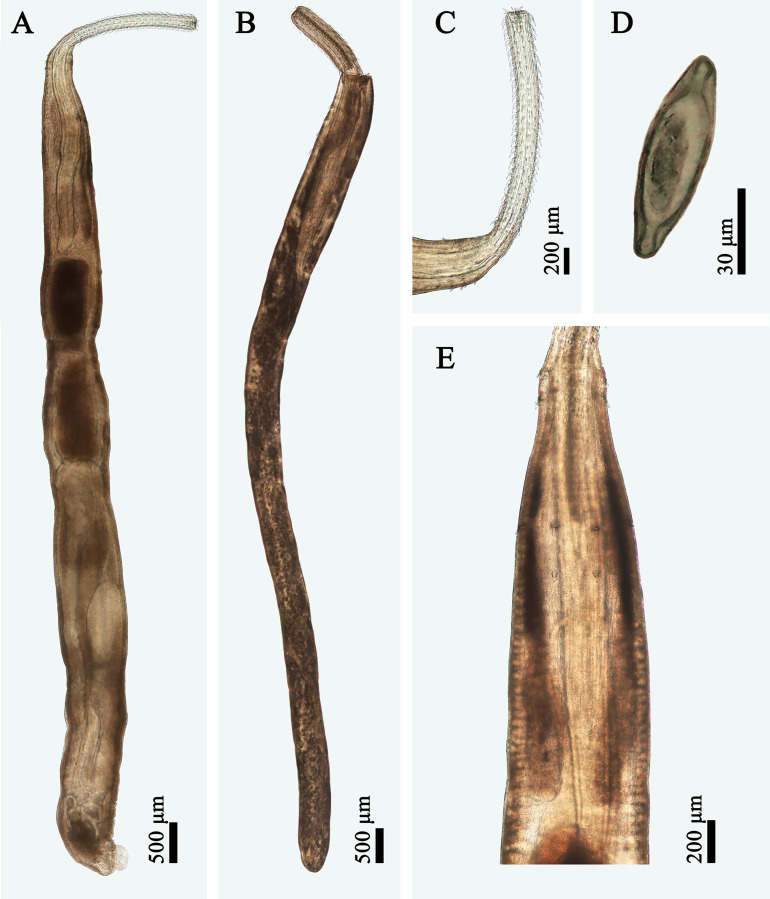
Photomicrographs of morphotype I of *Rhadinorhynchus cololabis*. (A) Male, lateral view; (B) Female, lateral view; (C) Proboscis of male, lateral view; (D) Egg (isolated from body cavity); (E) Anterior part of male, lateral view.

**Figure 3:**
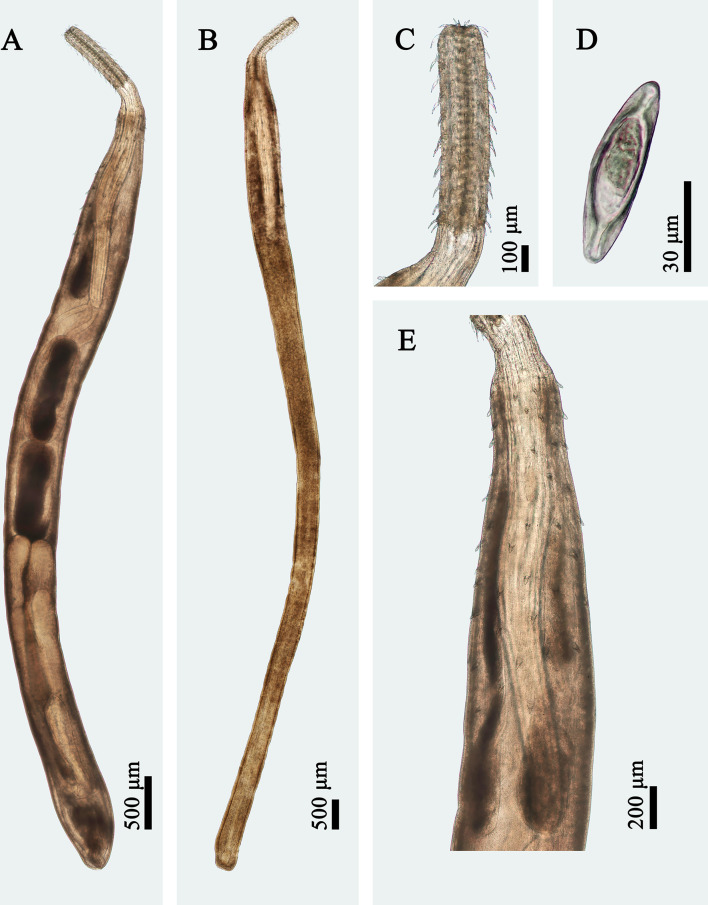
Photomicrographs of morphotype II of *Rhadinorhynchus cololabis* (proboscis not fully evaginated). (A) Male, lateral view; (B) Female, lateral view; (C) Proboscis of male, lateral view; (D) Egg (isolated from body cavity); (E) Anterior part of male, lateral view.

According to the number and distribution pattern of trunk spines, the present specimens were divided into two morphotypes (Figs. 1–3, Table 1). Morphometric comparison of the two different morphotypes of *R. cololabis* are provided in Table 2. Voucher specimens (morphotype I: HBNU–A–F20250710WL, morphotype II: HBNU–A–F20250711WL) were deposited in the College of Life Sciences, Hebei Normal University, Hebei Province, China.

**Table 1:** Two morphotypes of *Rhadinorhynchus cololabis* selected for molecular analysis.

	Samples	Characteristics	GenBank nos.
Morphotype I	5 males	Anterior trunk possessing fewer spines divided by a distinct aspinose zone into anterior and posterior fields in both sexes.	ITS: PV916386–PV916392
	2 females		*cox1*: PV864919–PV864925
			*cox2*: PV883210–PV883216
			12S: PV873439–PV873445
Morphotype II	2 males	Anterior trunk possessing more spines, 1–2 lateral spines connecting the anterior and posterior fields together (lacking a distinct aspinose zone) in both sexes.	ITS: PV916393–PV916398
	4 females		*cox1*: PV864926–PV864931
			*cox2*: PV883217–PV883222
			12S: PV873446–PV873451

**Table 2:** Morphometric comparisons of *Rhadinorhynchus cololabis*. *Abbreviations*: TL, trunk length; SP, size of proboscis; NRP, number of longitudinal rows of proboscis hooks; NHPR, number of hooks per longitudinal row; SAS, size of anterior trunk spines; SPS, size of posterior trunk spines; SPR, size of proboscis receptacle; SAT, size of anterior testis; SPT, size of posterior testis; LL, length of lemnisci; NCG, number of cement glands; SE, size of eggs. All measurements are in millimetres.

Source	Present study				Laurs & McCauley, 1964 [23]	
**Host**	*Cololabis saira*				*Cololabis saira*	
**Characteristics**	Morphotype I		Morphotype II			
	Male (*n* = 26)	Female (*n* = 4)	Male (*n* = 4)	Female (*n* = 23)	Male (*n* = 6)	Female (*n* = 6)
TL	5.77–11.1	9.78–26.6	7.67–9.09	9.76–24.69	9.20–12.2	18.0–26.7
SP	1.26–1.84 × 0.16–0.23	2.03–2.42 × 0.22–0.26	1.47–1.70 × 0.19–0.20	1.65–2.09 × 0.20–0.26	1.60–1.96 × 0.13–0.20	–
NRP	10–12	10–12	10–12	10–12	10	10–12
NHPR	20–22	20	20	20–21	20–21	–
SAS	0.028–0.068	0.039–0.068	0.034–0.048	0.034–0.077	0.028–0.046	0.041–0.059
SPS	0.033–0.072	0.058–0.092	0.044–0.063	0.053–0.089	0.034–0.051	0.060–0.074
SPR	2.15–3.02 × 0.24–0.37	2.66–4.12 × 0.27–0.42	2.36–2.63 × 0.23–0.32	2.93–3.97 × 0.24–0.38	2.40–3.14 × 0.23–0.30	2.32–3.50 × 0.26–0.33
LL	1.54–2.98	2.02–2.15	2.17–2.44	1.59–3.37	2.40–3.14	2.32–3.50
SAT	0.61–1.80 × 0.17–0.56	N/A	0.98–1.39 × 0.32–0.59	N/A	1.20–1.76 × 0.40–0.70	N/A
SPT	0.66–1.68 × 0.27–0.51	N/A	0.88–1.44 × 0.29–0.51	N/A	0.80–2.20 × 0.35–0.60	N/A
NCG	4	N/A	4	N/A	4	N/A
SE	N/A	0.048–0.073 × 0.015–0.039	N/A	0.034–0.097 × 0.01–0.02	N/A	0.057–0.075 × 0.009–0.014

### Molecular procedures

A total of 13 specimens representing two morphotypes were selected for molecular analysis (Table 1). The genomic DNA of acanthocephalans was extracted using a Magnetic Universal Genomic DNA Kit (DP705) [Sangon Biotech (Shanghai) Co., Ltd., Shanghai, China], according to the manufacturer’s instructions. The primers and cycling conditions used for amplifying the target sequences of ITS, *cox1*, *cox2*, and 12S are provided in Table 3 [15, 22]. PCR products were purified, sequenced, and analyzed according to methods reported previously [9, 27, 50, 51]. The sequence data obtained herein were deposited in GenBank (http://www.ncbi.nlm.nih.gov) under the accession numbers provided in Table 1.

**Table 3:** Primers and cycling conditions used for amplification of target regions of *Rhadinorhynchus cololabis*.

Primers	Primer sequences (5′–3′)	Target region	Cycling condition	Reference
ITS-F forward	5′–GTCGTAACAAGGTTTCCGTA–3′	ITS	94 °C for 2 min	[22]
ITS-R reverse	5′–TATGCTTAAATTCAGCGGGT–3′		94 °C for 20 s	
			51 °C for 20 s	
			65 °C for 50 s (40 cycles)	
			65 °C for 5 min	
*cox1*-F forward	5′–AGTTCTAATCATAA(R)GATAT(Y)GG–3′	*cox*1	94 °C for 5 min	[15]
*cox1*-R reverse	5′–TAAACTTCAGGGTGACCAAAAAATCA–3′		94 °C for 1 min	
			40 °C for 1 min	
			72 °C for 1 min (35 cycles)	
			72 °C for 5 min	
*cox2*-F forward	5′–GTGTTAAAGTGAGGTAGACTGG–3′	*cox*2	94 °C for 2 min	Present study
*cox2*-R reverse	5′–CTATGCATTACCCCACATAACTC–3′		94 °C for 30 s	
			52 °C for 30 s	
			72 °C for 30 s (33 cycles)	
			72 °C for 2 min	
12S-F forward	5′–CAGATTAAACTTGTGCCAGCGG–3′	12S	94 °C for 2 min	Present study
12S-R reverse	5′–CCCACGCTTAAAGCTACAACGAC–3′		94 °C for 30 s	
			58 °C for 30 s	
			72 °C for 30 s (33 cycles)	
			72 °C for 2 min	

### Species delimitation and phylogenetic analyses

ASAP analyses were used for species partition of different morphotypes of present samples and 10 species of *Rhadinorhynchus*, including *R. decapteri* Parukhin & Kovalenko, 1976, *R. dorsoventrospinosus* Amin, Heckmann & Ha, 2011, *R. hiansi* Soota & Bhattacharya, 1981, *R. johnstoni* Golvan, 1969, *R. laterospinosus*, *R. mariserpentis* (Steinauer *et al.*, 2019), *R. seriolae* (Yamaguti, 1963), *R. trachinoti* Grano-Maldonado *et al.*, 2025, *R. villalobosi* Martínez-Flores, García-Prieto & Oceguera-Figueroa, 2025 and *Rhadinorhynchus* sp., based on the partial nuclear ITS region, and mitochondrial *cox1*, *cox2*, and 12S sequence data, respectively. The ASAP analyses were executed using the ASAP online server (https://bioinfo.mnhn.fr/abi/public/asap/) under the Kimura (K80) ts/tv model. Among the recommended results by ASAP, the one with the lowest score was considered to be the optimal result according to the ASAP program [34].

BI analyses were performed to clarify the phylogenetic relationships of the present specimens and *Rhadinorhynchus* spp. using MrBayes 3.2.7 [36] with two parallel runs (2,000,000 generations) under the optimal models: GTR+F+G4 for *cox1*, HKY+F+I for *cox2*, HKY+F+I for 12S, and HKY+F for ITS. *Pseudoacanthocephalus sichuanensis* Zhao *et al.*, 2024 (Palaeacanthocephala: Echinorhynchida) was treated as the outgroup.

### Assembly and annotation of the mitogenome

A total of 50 Gb of clean genomic data were generated using the Pair-End 150 sequencing method on the Illumina NovaSeq 6000 platform by Novogene (Tianjin, China). The methods and procedures used for assembly, annotation and comparative analysis of the complete mitochondrial genome of *R. cololabis* are according to the previous studies [25, 45, 49–51], using the following software programs or tools, including GetOrganelle v1.7.2a [19], MitoS web server (http://mitos.bioinf.uni-leipzig.de/index.py), MitoZ v3.6, ORF finder web server (https://www.ncbi.nlm.nih.gov/orffinder/), ViennaRNA module [17], MitoS2 [6], RNAstructure v6.3 [35], Codon Adaptation Index (CAI) [24], and CGView online server V1.0 (http://stothard.afns.ualberta.ca/cgview_server/). The mitogenome of *R. cololabis* was deposited in GenBank (http://www.ncbi.nlm.nih.gov) under accession number PV866798.

## Results

### Molecular characterization

#### Partial ITS region

The 13 partial ITS sequences of *R. cololabis* obtained herein were all 546 bp in length, with no nucleotide divergence detected. There are only two *Rhadinorhynchus* species with ITS data registered in GenBank. Pairwise comparison of nucleotide differences in the ITS sequences of *R. cololabis* obtained herein with that of *Rhadinorhynchus* spp. available in GenBank ranged from 9.60% (*R. mariserpentis*, MK014834) to 15.3% (*R. dorsoventrospinosus*, MH384822).

#### Partial cox1 region

The 13 partial *cox1* sequences of *R. cololabis* obtained herein were all 655 bp in length, representing 13 different genotypes with 0.61–2.44% nucleotide divergence detected. There are 10 *Rhadinorhynchus* species with *cox1* data registered in GenBank. Pairwise comparison of nucleotide differences in the *cox1* sequences of *R. cololabis* with that of *Rhadinorhynchus* spp. available in GenBank ranged from 5.80% (*R. laterospinosus*, OR625531) to 26.5% (*R. decapteri*, KJ590125).

#### Partial cox2 region

The 13 partial *cox2* sequences of *R. cololabis* obtained herein were all 548 bp in length, representing 13 different genotypes with 0.18–2.55% nucleotide divergence detected. To date, only *R. laterospinosus* with the *cox2* sequence (PV590110) is available in GenBank. Pairwise comparison of nucleotide differences in the *cox2* sequences of *R. cololabis* with that of *R. laterospinosus* ranged from 3.83% to 4.93%.

#### Partial 12S region

The 13 partial 12S sequences of *R. cololabis* obtained herein were all 416 bp in length, representing 10 different genotypes with 0.24–1.68% nucleotide divergence detected. To date, only *R. laterospinosus* with the 12S sequence (PV590110) is available in GenBank. Pairwise comparison of nucleotide differences in the 12S sequences of *R. cololabis* with that of *R. laterospinosus* ranged from 2.21% to 3.19%.

### ASAP and BI analyses

**Figure 4:**
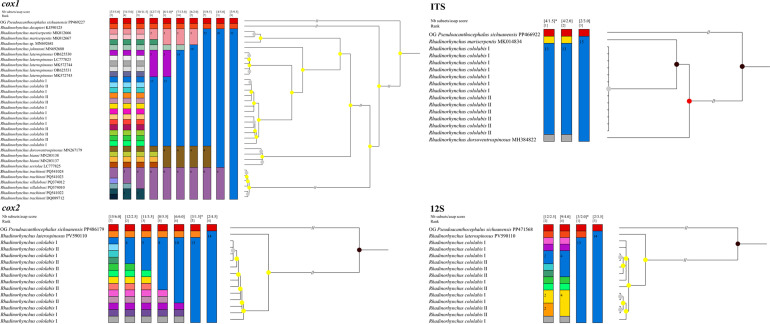
Assemble Species by Automatic Partitioning (ASAP) analyses of *Rhadinorhynchus* spp. conducted based on different nuclear and mitochondrial genetic markers. *Pseudoacanthocephalus sichuanensis* was chosen as the outgroup. The asterisk (*) indicates the best result according the lowest score and optimal recommendation by ASAP.

**Figure 5:**
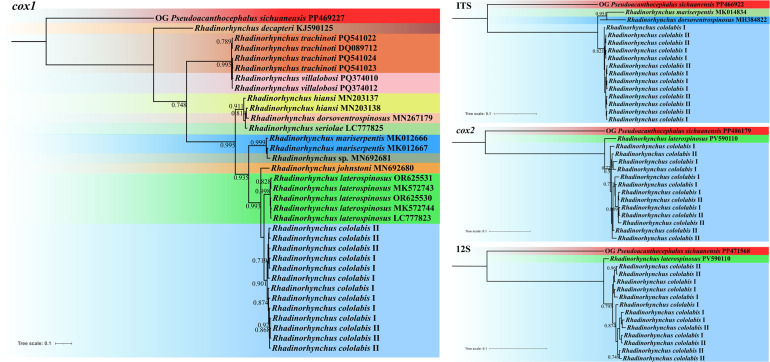
Bayesian inference (BI) results of *Rhadinorhynchus* spp. based on different nuclear and mitochondrial genetic markers. *Pseudoacanthocephalus sichuanensis* was chosen as the outgroup.

The present results of the ASAP analyses of the *cox1*, *cox2*, 12S, and ITS sequences all showed *R. cololabis* representing a distinct species from the other *Rhadinorhynchus* species (Fig. 4). Additionally, the ASAP result of *cox1* data did not support the current species partition of *R. seriolae*, *R. hiansi*, and *R. dorsoventrospinosus*, together with *R. villalobosi* and *R. trachinoti* (Fig. 4)*.* All the present BI results revealed *R. cololabis* forming a separate branch from the other *Rhadinorhynchus* species, but none of the results supported the current two morphotypes of *R. cololabis* belonging to distinct genetic lineages (different samples of the two morphotypes of *R. cololabis* mixed together) (Fig. 5). The BI using *cox1* data displayed samples of *R. villalobosi* nested into *R. trachinoti*, and *R. seriolae* and *R. dorsoventrospinosus* nested into *R. hiansi* (Fig. 5).

### Characterization of the mitogenome

**Figure 6:**
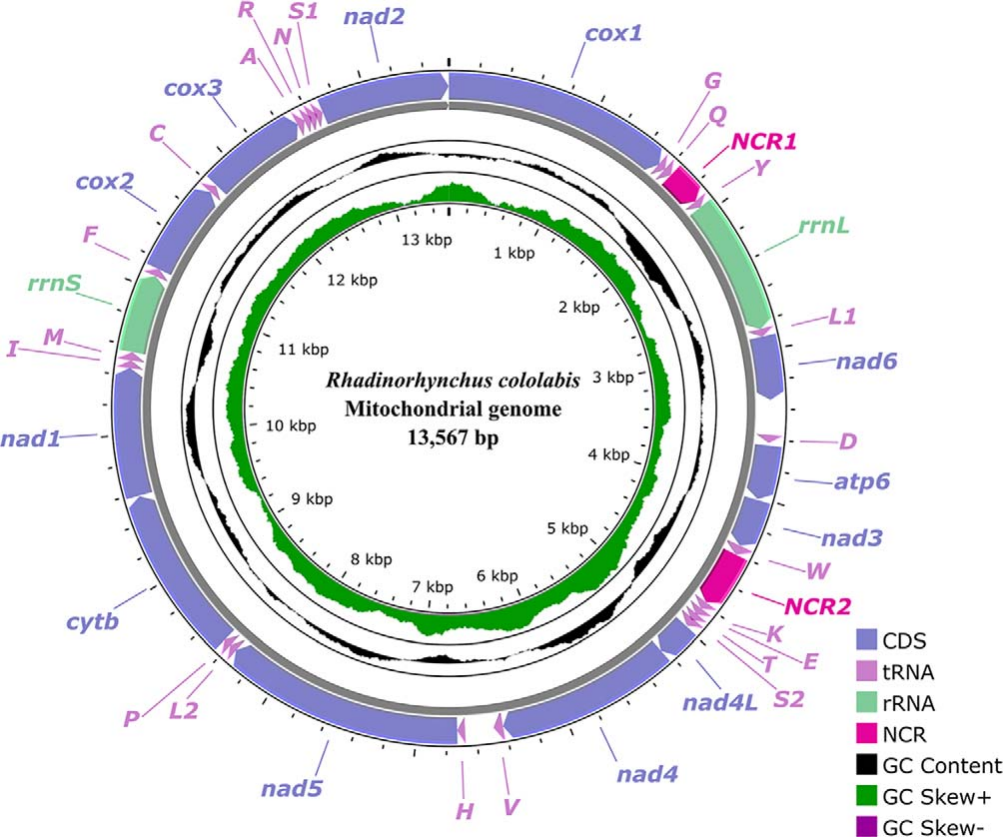
Gene map of mitochondrial genome of *Rhadinorhynchus cololabis.* All genes are transcribed in the clockwise direction on the same strand.

The complete mitogenome of *R. cololabis* is 13,567 bp in size, and includes 36 genes, containing 12 protein-coding genes (PCGs) (*cox1*–*3*, *cytb, nad1–6, nad4L* and *atp6*; missing *atp8*), 22 transfer RNA (tRNAs) genes, and 2 ribosomal RNA (rRNA) genes (*rrnL* is 916 bp, located between *tRNA-Tyr* and *tRNA-Leu1*; *rrnS* is 534 bp, located between *tRNA-Met* and *tRNA-Phe*), plus two non-coding regions (NCRs) (NCR1 is 242 bp, located between *tRNA-Gln* and *tRNA-Tyr*; NCR2 is 377 bp, located between *tRNA-Trp* and *tRNA-Lys*) (Fig. 6, Table 4). All genes are transcribed from the same strand in the same direction. The overall A+T content in the mitogenome of *R. cololabis* is 63.1%, displaying a strong nucleotide compositional bias toward A+T. The nucleotide content of the *R. cololabis* mitogenome is provided in Tables 4 and 5.

**Table 4:** Organization of the mitochondrial genome of *Rhadinorhynchus cololabis*.

Gene	Type	Start	End	Length (bp)	Start Codon	Stop Codon	Anticodon	Strand	Gap or Overlap
*cox1*	CDS	1	1539	1539	GTG	TAG		+	−2
*trnG*	tRNA	1538	1590	53			ucc	+	−2
*trnQ*	tRNA	1589	1645	57			uug	+	0
*NCR1*	Non-coding region	1646	1887	242				+	0
*trnY*	tRNA	1888	1938	51			gua	+	0
*rrnL*	rRNA	1939	2854	916				+	0
*trnL1*	tRNA	2855	2906	52			uag	+	0
*nad6*	CDS	2907	3335	429	GTG	TAG		+	236
*trnD*	tRNA	3572	3623	52			guc	+	19
*atp6*	CDS	3643	4008	366	ATG	TAG		+	7
*nad3*	CDS	4016	4343	328	TTG	T		+	0
*trnW*	tRNA	4344	4409	66			uca	+	0
*NCR2*	Non-coding region	4410	4786	377				+	0
*trnK*	tRNA	4787	4841	55			uuu	+	−1
*trnE*	tRNA	4841	4892	52			uuc	+	−4
*trnT*	tRNA	4889	4941	53			ugu	+	−14
*trnS2*	tRNA	4928	4988	61			uga	+	6
*nad4L*	CDS	4995	5231	237	TTG	TAA		+	−1
*nad4*	CDS	5231	6420	1190	ATG	TA		+	0
*trnV*	tRNA	6421	6484	64			uac	+	193
*trnH*	tRNA	6678	6729	52			gug	+	0
*nad5*	CDS	6730	8346	1617	GTG	TAG		+	−1
*trnL2*	tRNA	8346	8397	52			uaa	+	0
*trnP*	tRNA	8398	8448	51			ugg	+	0
*cytb*	CDS	8449	9579	1131	ATG	TAG		+	5
*nad1*	CDS	9585	10449	865	ATT	T		+	−2
*trnI*	tRNA	10448	10500	53			gau	+	−1
*trnM*	tRNA	10500	10556	57			cau	+	0
*rrnS*	rRNA	10557	11090	534				+	0
*trnF*	tRNA	11091	11143	53			gaa	+	−2
*cox2*	CDS	11142	11765	624	GTG	TAA		+	0
*trnC*	tRNA	11766	11818	53			gca	+	7
*cox3*	CDS	11826	12509	684	ATT	TAA		+	−2
*trnA*	tRNA	12508	12562	55			ugc	+	−2
*trnR*	tRNA	12561	12614	54			ucg	+	−17
*trnN*	tRNA	12598	12656	59			guu	+	−14
*trnS1*	tRNA	12643	12696	54			acu	+	0
*nad2*	CDS	12697	13566	870	TTG	TAG		+	1

**Table 5:** Base composition and skewness of *Rhadinorhynchus cololabis*.

Location/Species	A%	T%	C%	G%	AT%	AT-skew	GC-skew	Total (bp)
Whole mitochondrial genome	24.16	38.95	10.54	26.35	63.11	−0.23	0.43	13,567
Protein coding genes (PCGs)	22.07	39.38	10.68	27.86	61.46	−0.28	0.45	9,880
1st codon	24.13	30.08	10.20	35.60	54.20	−0.11	0.55	3,295
2nd codon	13.88	48.10	12.94	25.08	61.98	−0.55	0.32	3,293
3rd codon	28.22	39.98	8.90	22.90	68.20	−0.17	0.44	3,292
tRNAs	27.79	38.79	10.17	23.24	66.58	−0.17	0.39	1,209
rRNAs	32.34	35.72	10.28	21.66	68.07	−0.05	0.36	1,450
*rrnL*	32.42	35.81	10.48	21.29	68.23	−0.05	0.34	916
*rrnS*	32.21	35.58	9.93	22.28	67.79	−0.05	0.38	534
Non-coding region 1	27.27	46.69	7.02	19.01	73.97	−0.26	0.46	242
Non-coding region 2	33.16	37.14	9.81	19.89	70.29	−0.06	0.34	377

**Figure 7:**
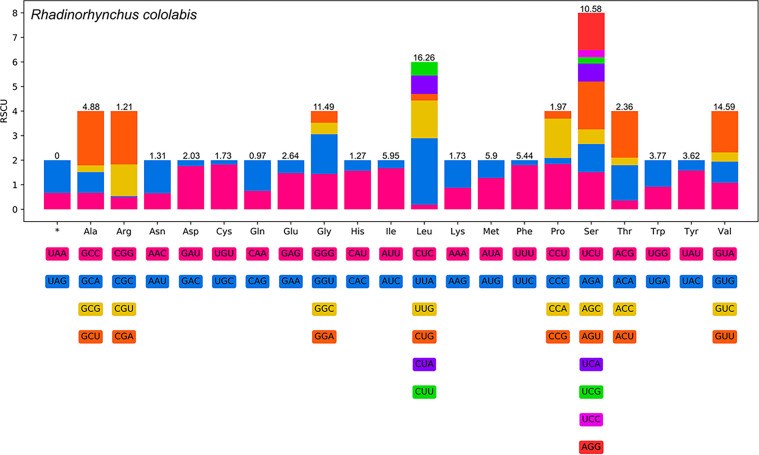
Relative synonymous codon usage (RSCU) of *Rhadinorhynchus cololabis*. Codon families (in alphabetical order, from left to right) are provided below the horizontal axis. Values on top of each bar represent amino acid usage in percentage.

**Figure S1:**
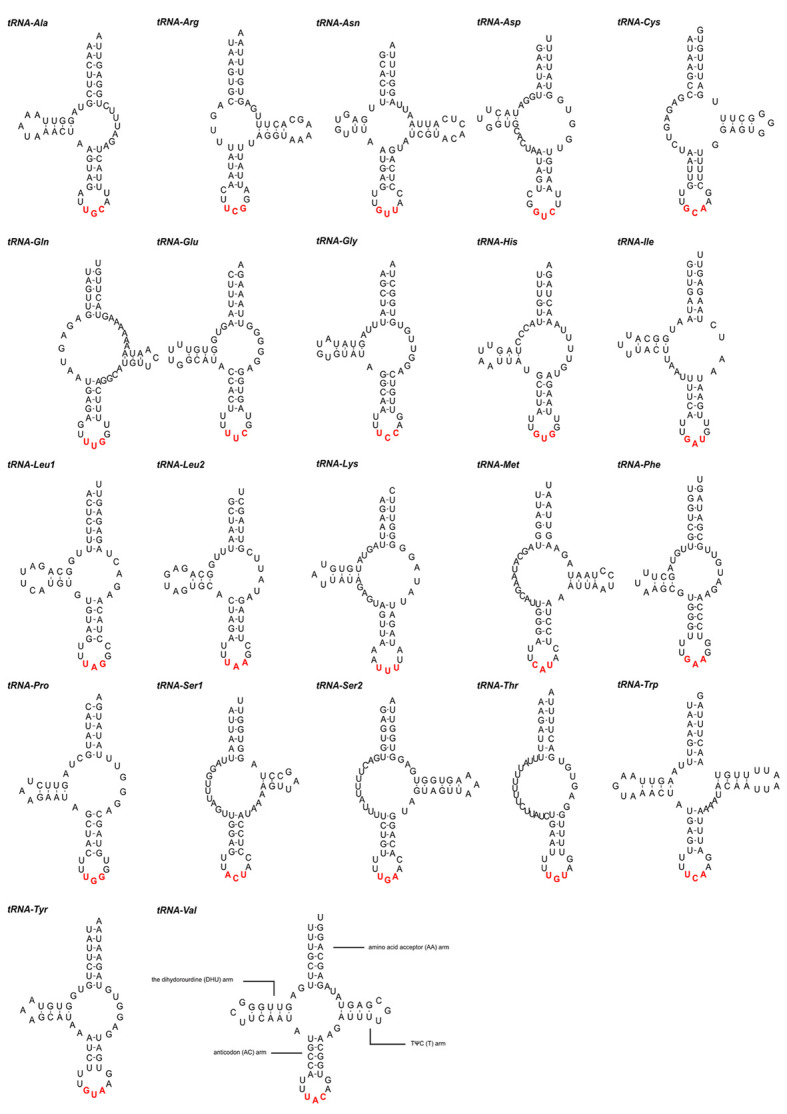
The predicted secondary structures of 22 tRNAs in the mitogenome of *Rhadinorhynchus cololabis* (Watson-Crick bonds indicated by lines, GU bonds indicated by dots, grey bold bases representing anticodons). The tRNAs are labelled with the abbreviations of their corresponding amino acids according to IUPAC-IUB code.

The total size of the 12 PCGs of the present mitogenome is 9,880 bp (excluding stop codons), varied from 237 bp (*nad4L*) to 1617 bp (*nad5*) for each gene, which encode 3,292 amino acids. Among the 12 PCGs, four genes (*cox1*, *cox2*, *nad5*, and *nad6*) use GTG as the start codon, whereas three genes (*atp6*, *nad4*, and *cytb*) use ATG, and the other three genes (*nad2*, *nad3*, and *nad4L)* use TTG. The remaining two genes, *cox3* and *nad1*, use ATT as the start codon. TAG is the most commonly used stop codon for 6 genes (*cox1*, *nad2*, *nad5*, *nad6*, *atp6*, and *cytb*). The remaining three genes (*cox2*, *cox3*, and *nad4L*) use TAA as the stop codon. The incomplete stop codon T is inferred for two genes (*nad1* and *nad3*), while only *nad4* uses the incomplete stop codon TA (Table 4). Detailed information on overall codon usage and RSCU for the 12 PCGs is shown in Figure 7. A total of 22 tRNAs were identified with lengths ranging from 51 bp (*trnY* and *trnP*) to 66 bp (*trnW*). The lengths of 22 tRNAs and their anticodon secondary structures are provided in Table 4 and Supplementary file: Figure S1.

**Figure S2:**
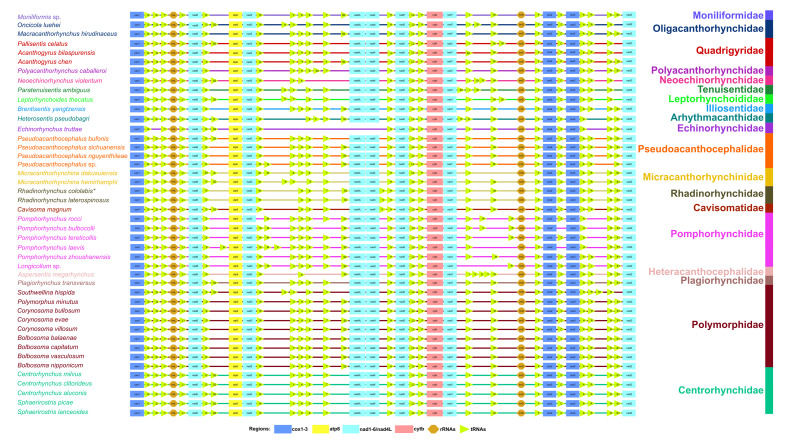
Comparison of the linearized gene arrangement of acanthocephalan mitogenomes. All genes are transcribed in the same direction from left to right. The tRNAs are labelled by a single-letter code for the corresponding amino acid. The non-coding regions are not included*. Rhadinorhynchus cololabis* indicated using an asterisk (*).

The gene arrangement of the 36 genes in the mitogenome of *R. cololabis* is in the following order: *cox1*, *tRNA-Gly*, *tRNA-Gln*, *tRNA-Tyr*, *rrnL, tRNA-Leu1*, *nad6*, *tRNA-Asp*, *atp6*, *nad3*, *tRNA-Trp*, *tRNA-Lys*, *tRNA-Glu*, *tRNA-Thr*, *tRNA-Ser2*, *nad4L*, *nad4, tRNA-Val*, *tRNA-His*, *nad5*, *tRNA-Leu2*, *tRNA-Pro*, *cytb*, *nad1*, *tRNA-Ile*, *tRNA-Met*, *rrnS*, *tRNA-Phe*, *cox2*, *tRNA-Cys*, *cox3*, *tRNA-Ala*, *tRNA-Arg*, *tRNA-Asn*, *tRNA-Ser1*, and *nad2* (Fig. 6 and Supplementary file: Fig. S2).

## Discussion

Laurs & McCauley (1964) [23] originally described *R. cololabis* from *C. saira* from waters off Oregon, United States, with a description of *R. cololabis* that is rather good for its time. Subsequently, Motora (2016) [32] found *R. cololabis* from *O. masou* in the Sea of Japan. This study is the first record of this species in Chinese waters. The morphology and measurements of our specimens are almost identical to the original description of *R. cololabis*, including the trunk length, the size, shape and armature of the proboscis, the size of the proboscis receptacle and testis, the shape and size of the trunk spines, the number of cement glands, and the morphology and size of eggs (Table 2). Additionally, the present specimens were also collected from the type host *C. saira*. Consequently, we have no hesitation in considering the present material being conspecific with *R. cololabis*.

In this study, striking variability in the number and distribution of trunk spines was observed among different individuals of *R. cololabis* for the first time. According to the number and distribution of trunk spines (presence or absence of a distinct aspinose zone separating the trunk spines into anterior and posterior fields), the present specimens were divided into two distinct morphotypes, which might be treated as separate taxa under the traditional morphospecies concept. However, molecular analysis of the nuclear ITS region showed no nucleotide divergence among different individuals of the two morphotypes. By contrast, the mitochondrial sequence data displayed a relatively broad range of intraspecific nucleotide divergence among different individuals of *R. cololabis* (0.61–2.44%, 0.18–2.55%, and 0.24–1.68% detected in the *cox1*, *cox2*, and 12S, respectively). Additionally, the ASAP and BI results using different sequence data all revealed that the two distinct morphotypes of *R. cololabis* are conspecific and do not represent two separate genetic lineages.

Recent molecular phylogenetic studies revealed that *R. seriolae*, *R. hiansi*, and *R. dorsoventrospinosus* have very close relationships within *Rhadinorhynchus* [2, 21, 31, 40, 48]. However, the present ASAP and BI analyses of *cox1* data did not support the current species partition of *R. hiansi*, *R. dorsoventrospinosus*, and *R. seriolae*. The validity of *R. hiansi* and *R. dorsoventrospinosus* were challenged. In fact, *R. seriolae* and *R. dorsoventrospinosus* both mainly parasitize fishes of the Scombridae and Carangidae, which also share the same or similar distribution regions, and have similar morphological characters, except for different number and distribution of trunk spines [2, 8, 21, 38, 48]. Additionally, some previous studies reported the presence of a broad range of variation in the number and distribution of trunk spines in *R. seriolae* [21, 38]. Consequently, we speculate that *R. dorsoventrospinosus* simply represents a particular morphotype of *R. seriolae*, and suggest to list it as a junior synonym of *R. seriolae*. However, the type host of *R. hiansi* is *Ablennes hians* Valenciennes (Beloniformes: Belonidae), and this species has 21–24 longitudinal alternating rows of 36–48 hooks each, which are distinctly different from those of *R. seriolae*. The taxonomical status of *R. hiansi* needs further study in the future.

Grano-Maldonado *et al.* (2025) [16] described the adult of *R. trachinoti* from the Gafftopsail pompano *Trachinotus rhodopus* Gill (Carangiformes: Carangidae) from off the coast of Mazatlán, Mexico and the cystacanth from the mysid crustacean, *Metamysidopsis frankfiersi* (Hendrickx & Hernández-Payán) from Playa Novillero, Mexico. Later, Martínez-Flores *et al.* (2025) [31] described *R. villalobosi* from the same definitive host of *R. trachinoti*. These authors mentioned that *R. villalobosi* differs from *R. trachinoti* on the number of trunk spines, position of the genital pore, and size of the cement glands. However, our ASAP and BI results of *cox1* data did not support that *R. villalobosi* and *R. trachinoti* represent two distinct species*.* In fact, the measurements of cement glands provided by Martínez-Flores *et al.* (2025) [31] seem to be erroneous and are not concordant with the illustration. The differences in the number of trunk spines and the position of genital pores between the two species should belong to intraspecific variation. Consequently, we suggest to treat *R. villalobosi* as a new synonym of *R. trachinoti.* The present findings also indicate that the number and distribution of trunk spines vary markedly in some cases of *Rhadinorhynchus*, and care must be taken when differentiating *Rhadinorhynchus* species using this feature.

In the Rhadinorhynchidae, only *R. laterospinosus* had its complete mitochondrial genome sequenced prior to this study [47]. The size of the mitogenomes of *R. cololabis* and *R. laterospinosus* are both 13,567 bp, which are the smallest among the reported mitogenomes of Echinorhynchida [10, 11, 33, 39, 41, 44–47, 49–51]. The 36 gene arrangement order of mitogenomes of *R. cololabis* and *R. laterospinosus* are also identical, which are different from those of the known mitogenomes of Acanthocephala so far [47]. Comparative mitogenomic analysis of *R. laterospinosus* and *R. cololabis* also displayed a low level of divergence in both nucleotide sequences of whole mitogenomes (5.40%) and amino acid sequences of 12 protein-coding genes (PCGs) (6.20%), which are distinctly higher than the intraspecific variation level of mitogenome of *Pomphorhynchus pagrosomi* (*vs* nucleotide sequences = 2.40%, amino acid sequences = 1.20%) [51]. Consequently, comparative mitogenomics also supported that *R. cololabis* and *R. laterospinosus* belong to two separate taxa.

## Conclusions

In this study, we found an example of phenotypic variation in trunk spines of the poorly known rhadinorhynchid species *R. cololabis* collected from *C. saira* in the South China Sea. According to the number and distribution of trunk spines, the present specimens of *R. cololabis* can be divided into two distinct morphotypes, which may erroneously be recognized as distinct taxa in the absence of molecular data. However, the ASAP and BI results using different nuclear and mitochondrial sequence data all confirm that the two distinct morphotypes of *R. cololabis* are conspecific, and do not represent two separate genetic lineages. Our ASAP and BI results of *cox1* data also question the validity of *R. villalobosi*, *R. hiansi*, and *R. dorsoventrospinosus*, and suggest to treat *R. villalobosi* as a synonym of *R. trachinoti*. The present findings also indicate that the number and distribution of trunk spines vary markedly in some cases, and care must be taken when differentiating *Rhadinorhynchus* species using this feature. Additionally, the complete mitogenome of *R. cololabis* is present for the first time, and displays a very high level of similarity with the mitogenome of *R. laterospinosus* in both nucleotide sequences (94.6%) and amino acid sequences of 12 protein-coding genes (93.8%). The results of ASAP, BI analyses, and comparative mitogenomics all support *R. cololabis* and *R. laterospinosus* representing two separate taxa.

## Data Availability

The datasets generated in the present study are available in the GenBank repository.

## Supplementary materials

The supplementary material of this article is available at https://www.parasite-journal.org/10.1051/parasite/2025069/olm.
